# Moving the boundaries to the South-East: first record of autochthonous *Angiostrongylus vasorum* infection in a dog in Vojvodina province, northern Serbia

**DOI:** 10.1186/1756-3305-7-396

**Published:** 2014-08-27

**Authors:** Stanislav Simin, Ljubica Spasojević Kosić, Ljiljana Kuruca, Ivan Pavlović, Milan Savović, Vesna Lalošević

**Affiliations:** Faculty of Agriculture, Department of Veterinary Medicine, University of Novi Sad, Trg Dositeja Obradovića 8, 21000 Novi Sad, Serbia; Scientific Veterinary Institute of Serbia, Vojvode Toze 14, 11000 Belgrade, Serbia; PVP MSV Medicus D.O.O, Milice Stojadinović Srpkinje 1, 21209 Bukovac, Serbia

**Keywords:** *Angiostrongylus vasorum*, Dog, Autochthonous infection, Emerging disease, Serbia

## Abstract

**Background:**

*Angiostrongylus vasorum* is a cardiopulmonary canine nematode, potentially fatal to its host. In the last decade, there has been an increasing number of autochthonous cases in areas previously considered non-endemic. However, information about the parasite’s occurrence and distribution among Central and Eastern (Southeastern) European countries are scarce. This paper reports the first recorded case of autochthonous *A. vasorum* infection in a hunting dog from Serbia.

**Findings:**

In March 2013, a female hunting dog was presented to a veterinary clinic in Novi Sad, Serbia, for examination of a chronic skin problem. The dog had no history of respiratory or cardiovascular diseases. Faecal and urine samples were collected and examined for the presence of parasite ova/cysts. A modified Baermann test detected 8.8 larvae per gram of faeces. Based on their overall body length (mean 381.7 ± 15.9 μm; range from 342.5 to 404.3 μm; n = 12) and characteristic tail morphology, they were identified as the first-stage larvae of *A. vasorum*.

**Conclusions:**

The spread of *A. vasorum* to the southeast of Europe is further confirmed after finding autochthonous infected dog from Serbia. Therefore, veterinary professionals in Serbia should consider *A. vasorum* in differential diagnosis of dogs.

## Findings

### Background

*Angiostrongylus vasorum* (the French heartworm) is a metastrongylid nematode that resides in the right chamber of the heart and pulmonary arteries of domestic dogs (*Canis familiaris*), wild canids (primarily red fox (*Vulpes vulpes*)) and occasionally other animals [1]. The life cycle is indirect and definitive hosts are infected after ingestion of infective larvae (L3) located in tissues of terrestrial and aquatic snails and slugs (intermediate hosts) or frogs (both intermediate and paratenic hosts, as confirmed experimentally) [2]. In dogs, the infection can be asymptomatic or accompanied with variable clinical symptoms. Most commonly, the signs are cardiorespiratory in nature, followed by coagulopathies and neurological signs. These symptoms may occur either singly or in combination, potentially leading to sudden death of the host [3].

The parasite was first discovered by Serres in 1853 [4] from the right side of the heart and pulmonary artery of a 2 year old pointer dog in Toulouse, Southern France. Since then, *A. vasorum* has been reported in Europe, Africa, North and South America. In Europe, traditionally endemic foci are located in western countries (e.g. in France, Denmark, southern Britain, Ireland), but due to recent emergence of *A. vasorum*, autochthonous cases have been recorded in various parts of the continent. Still, information about the parasite’s occurrence and distribution among central and eastern (Southeastern) European countries is scarce [5, 6]. In this region, *A. vasorum* has been recorded in dogs and/or foxes in Croatia [7], Greece [8], Hungary [9, 10], Poland [6] and Slovakia [5].

This paper reports the first recorded case of autochthonous *A. vasorum* infection in a dog from Serbia.

## Methods

### Case presentation

In March 2013, a seven year old intact female Posavac hound was presented to a veterinary clinic in Novi Sad, for examination of a chronic skin problem. The dog originated from Sremska Kamenica (45°13′14″N, 19°50′21″E), Vojvodina province (Northern Serbia), had never left the country, but was taken for wild boar hunting inside the province. The owner reported regular annual vaccination and prophylactic anthelmintic treatment at three month intervals using a combination of praziquantel, pyrantel embonate and febantel (Drontal Plus tablets, Bayer). Parasitological examination had not been performed.

According to the owner, the dog had no history of respiratory or cardiovascular diseases. Physical examination revealed normal temperature, mild lymphadenopathy and pruritic skin lesions located on all four extremities, muzzle, ventral neck and abdomen. Prior to examination, the dog had been treated with subcutaneous (SC) ivermectin by a veterinarian who had suspected sarcoptic mange, but the manufacturer, dosage and the frequency of the treatment were not ascertainable. Skin scrapings were negative for the presence of canine ectoparasites; cytological examination of skin lesions was not performed. Fresh samples of faeces and urine were collected for parasitological examination.

### Parasitological examination

Samples were examined by centrifugal faecal flotation (Sheater’s sugar solution, specific gravity 1.27) and urine sedimentation [11], for the presence of parasite ova/cysts.

A faecal sample was analysed by a modified Baermann test [11]. Larvae found in suspension were morphologically consistent with *A. vasorum*. For detailed morphologic and morphometric analysis, twelve larvae, killed with Lugol’s iodine, were examined, imaged and measured using a Leica DMLS light microscope (Leica Microsystems, Wetzlar, Germany) at × 400 magnification. The first-stage larvae of *A. vasorum* were identified by their length and tail morphology according to available keys [11, 12].

### Case follow up

The owner was advised to treat the dog with fenbendazole (50 mg/kg body weight for 14 days [13]). Unfortunately, no control of the recommended therapy or additional diagnostic procedures (such as radiography) was performed, due to the disinterest of the owner.

## Results and discussion

**Figure 1:**
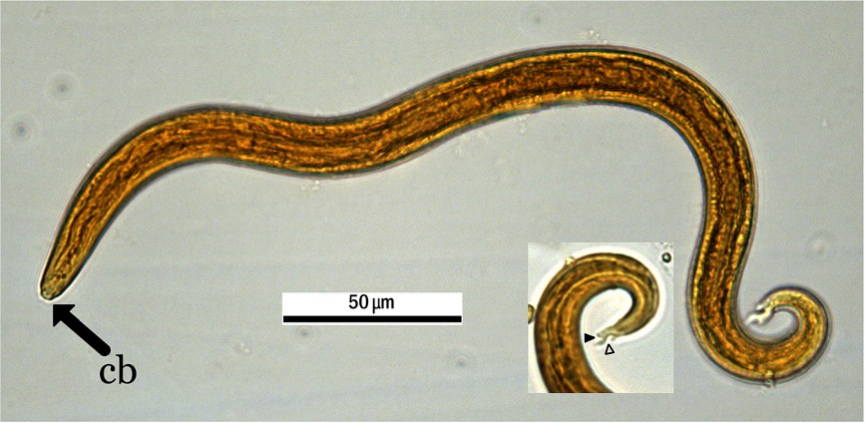
**Morphology of the first stage larva of** ***Angiostrongylus vasorum*** **.** First stage larvae were isolated using a modified Baermann technique and killed with Lugol’s iodine. Note tip of the tail with dorsal indentation (outlined white arrowhead) and ventral indentation (black arrowhead), as diagnostic morphological characteristics, and cephalic button (cb) located at anterior end.

No parasite eggs/cysts were recovered after faecal flotation and urine sedimentation. Detailed analysis of the larvae enabled clear visualisation of tail morphology; the tip, with characteristic dorsal and ventral indentation, could be readily seen (Figure 1). The larvae were alive and motile when transferred to slides; the dead larvae were coiled, C shaped or S shaped. Most of the observed larvae had a cephalic button at the anterior end. Mean body length was (μm ± SD) 381.7 ± 15.9; range: 342.5 to 404.3 μm (n = 12). Based on the overall length of the larvae and tail morphology, they were identified as the first-stage larvae of *A. vasorum*, with a larval load of 8.8 larvae per gram of faeces.

To our knowledge, the current report is the first reported case of autochthonous *A. vasorum* infection in a dog from Serbia. This finding, together with other recent reports of the parasite in dogs and foxes from European countries east of Germany and Italy, provides important evidence that the parasite’s range is expanding further to the southeast.

According to available data, Serbia is abundant with terrestrial and aquatic gastropods [14–16], and the climate in Vojvodina [17] offers suitable conditions for their survival. Among the gastropod species present, we have found at least ten proven to be either natural or experimental hosts of *A. vasorum*.

It is assumed that the dog acquired *A. vasorum* via infected gastropods from its surrounding home environment, or during hunting. A source of L1 for the resident mollusc population could be the red fox, as a natural host and an important reservoir of this parasite. *A. vasorum* is still not confirmed in foxes in Serbia, but it is present in neighbouring countries [7, 9]. This could have resulted in introduction of the parasite to Serbia, since proliferation and increased movement of this carnivore is evident across Europe [18].

Based on anamnesis, the dog from our study did not show any symptoms related to angiostrongylosis, although a display of discrete clinical signs could have passed unnoticed by the owner. It is not unusual for dogs to be subclinically infected, as shown in previous reports [e.g. 5, 10]. The duration of infection of this dog is unknown, although the asymptomatic course may indicate an early stage of infection [19]. It is important to find and treat subclinically infected dogs, since sudden death, mostly due coagulopathies [3], may occur in untreated chronic infections.

A number of different techniques are available for diagnosis of *A. vasorum* [see e.g. 1]. In the current study, L1 of *A. vasorum* were detected using the Baermann test, which is widely used as a simple, cost-effective and relatively rapid diagnostic method [3, 5]. Another important advantage of this test is the ability to rule out other lungworms, since characteristic tail morphology and length of the larvae of *A. vasorum* are diagnostic features [12]. On the other hand, the method has several limitations including inability to diagnose infection during the prepatent period, the requirement for well-trained personnel [5], and more importantly, false negative results due to intermittent shedding of L1, despite the presence of clinical symptoms [19]. Examination of faecal samples over three consecutive days in order to increase the sensitivity of Baermann technique is recommended [3].

The combination of praziquantel, pyrantel embonate and febantel is inappropriate to treat *A. vasorum*, as illustrated by the finding of L1 in the faeces of dogs from Ireland [20] and in the dog from our study, which had been treated with this drug combination. Several drugs are available for effective treatment of *A. vasorum,* such as fenbendazole, milbemycin oxime (administered orally) and imidacloprid/moxidectin (topical) [21–23]. It should be noted that despite successful treatment, larval excretion may continue for over three weeks [24].

Besides its importance in control of therapy success, Baermann examination is still valuable for routine screening of *A. vasorum* infection, for the reasons given above. Alternatively, serological tests (for antigen and antibody detection) [25, 26] and in-clinic test kits [27] are now available for early and correct diagnosis. This enables early treatment of dogs, which leads to a better outcome and prognosis.

## Conclusions

This study presented the first case of autochthonous *A. vasorum* infection in a dog in Serbia, confirming the spread of this parasite to this part of South-Eastern Europe. Therefore, veterinary professionals in Serbia should be aware of the presence of this parasite and consider *A. vasorum* during differential diagnosis of dogs.
